# Spatial Distribution of Hunting Billbugs (Coleoptera: Curculionidae) in Sod Farms

**DOI:** 10.3390/insects12050402

**Published:** 2021-04-30

**Authors:** Midhula Gireesh, Jhalendra P. Rijal, Shimat V. Joseph

**Affiliations:** 1 Department of Entomology, University of Georgia, 1109 Experiment Street, Griffin, GA 30223, USA; svjoseph@uga.edu; 2 UC Statewide IPM Program, University of California Agriculture and Natural Resources, 3800 Cornucopia Way, Modesto, CA 95358, USA; jrijal@ucanr.edu

**Keywords:** *Sphenophorus* spp., turfgrass, sampling plan, IPM, SADIE, variogram

## Abstract

**Simple Summary:**

The hunting billbug is the most dominant and damaging insect pest species of sod farms (where turfgrass is commercially produced) in Georgia (USA). The larvae feed within the turfgrass stem, and roots affect turfgrass growth. Hunting billbugs are usually managed using insecticides. However, the application of insecticides to entire sod fields is not an economically and practically feasible option. Thus, an improved sampling plan for larvae and adults is warranted to improve management decisions. The current study was aimed at understanding the spatial distributions of hunting billbug larvae and adults in sod farms using geospatial techniques. The larvae and adults were sampled using soil cores and pitfall traps, respectively. After evaluating two geospatial techniques, the distribution pattern of hunting billbug larvae and adults within the sod farms was aggregated. The presence of billbugs in samples collected at 4 m apart suggests active infestation. This information will help develop integrated pest management for hunting billbug in sod farms and reduce insecticide use, benefiting growers and the environment alike.

**Abstract:**

The hunting billbug, *Sphenophorus venatus vestitus* Chittenden (Coleoptera: Curculionidae), is an important turfgrass pest, especially in sod farms. *S. venatus vestitus* larvae feed on the stems and roots of turfgrass. Damaged turfgrass is loosely held together and poses a challenge for machine harvesting. Additionally, the normal growth of turfgrass is affected, especially after winter dormancy. Because *S. venatus vestitus* larvae are hidden inside the stems or under the soil, larval management is challenging. To improve sampling and management, the spatial distribution patterns of *S. venatus vestitus* larvae and adults were assessed at four sod farm sites with a history of *S. venatus vestitus* infestation in central Georgia (USA). The larvae were sampled by soil cores using a hole cutter, whereas adults were collected using pitfall traps for 7 d. The spatial distributions of larvae and adults was analyzed using SADIE and variograms. The SADIE and variogram analyses revealed a significant aggregation pattern for adults, whereas aggregated distributions were detected for larvae with variogram analyses. The average ranges of spatial dependence for larval and adult samples were 3.9 m and 5.4 m, respectively. Interpolated distribution maps were created to visually depict *S. venatus vestitus* infestation hotspots within the sod farms.

## 1. Introduction

The hunting billbug, *Sphenophorus venatus vestitus* Chittenden (Coleoptera: Curculionidae), is a serious pest of warm-season turfgrass in the USA [1]. In Georgia, bermudagrasses (*Cynodon dactylon* (L). Pers), zoysiagrass (*Zoysia* spp.), St. Augustinegrass (*Stenotaphrum secondatum* (Walter) Kuntze), bahiagrass (*Paspalum notatum* Flugge), and centipedegrass (*Eremochloa ophiuroides* (Munro) Hack) are the major warm-season grasses and are produced on sod farms. These turfgrasses are grown over approximately 10,785 ha across 64 of 159 counties and are valued at $118 million USD [2]. *S. venatus vestitus* is present at high densities in Georgia sod farms [3]. Females prefer actively growing, thick stolons for oviposition, as eggs are inserted into the stolon [1]. The first instars feed within stems, and the late instar larvae leave the stolon and consume the roots [4]. The larvae go through five instars before pupating in the soil. Adults overwinter in protected areas of the soil, although the larval stages are also found in the soil during winter months [1]. In central Georgia, the adults emerge from the overwintering sites beginning in late winter, while overwintering larvae continue to develop in spring, and those adults emerge in late spring [3].

The damage and problem from *S. venatus vestitus* feeding develop differently in various commercial turfgrass settings. In golf courses, because the adults and larvae of *S. venatus vestitus* consume on the roots, injury symptoms initially appear as chlorosis. Over time, the affected turfgrass develops brown patches [5]. However, in sod farms, injury symptoms are rarely manifested because the sod is harvested rather quickly, e.g., within 1.5 years. Instead, the injured stolons and roots disintegrate during machine harvesting and pose a considerable challenge to growers (C. Carter, personal communication). In addition, the stress from *S. venatus vestitus* feeding and oviposition affects the normal growth and development of zoysiagrass, especially when the grass breaks winter dormancy in spring [1]. The slow growth habit of zoysiagrass may contribute to the population density of *S. venatus vestitus* in sod farms. Any delay in the growth and development of zoysiagrass poses an economic challenge to growers, as the sod is not delivered at the scheduled times.

Implementation of a successful integrated pest management (IPM) program relies on determining population thresholds by using reliable pest monitoring tools [6,7]. Mean- and variance-based models can be used to develop sampling plans for several arthropod pests [8,9,10], but these models only use the frequency distributions of pest counts without considering the spatial locations of the pest population samples. Therefore, these models are not suitable for characterizing within-field population distributions or for developing sampling plans [11,12]. Due to the lack of two-dimensional information for individual sample locations, the information derived from these mean-variance methods lacks many ecological interactions [13,14]. Another benefit of spatial distribution sampling is to develop a visual representation of pest infestations in the field by creating prediction maps and kriging maps in variograms [6,15,16] and “red and blue” maps in SADIE [17,18]. This type of visual representation can be useful for site-specific pest management efforts.

There are many examples of how spatial distribution information can be used to understand the ecology and management of arthropod pests. A spatial distribution study of the annual bluegrass weevil *Listronotus maculicollis* Dietz (Coleoptera: Curculionidae) in golf courses showed aggregates of adults and larvae along the edges of fairways [19]. Additionally, this study suggested that *L. maculicollis* can spread to entire golf courses from an initially aggregated colonization. Similarly, previous studies have developed an efficient and quantitative sampling strategy to assess grape root borer, *Vitacea polistiformis* (Harris), (Lepidoptera: Sesiidae) infestations in Virginia vineyards [11]; alfalfa weevil, *Hypera postica* (Gyllenhal) and their natural predators; (*Coccinella septempunctata* L., *Adalia bipunctata* L., *Nabis americoferus* Carayon and *N. ferus* L.) [20]; Kudzu bug, *Megacopta cribraria* [F.] and their egg parasitoid *Paratelenomus saccharalis* Dodd (Hymenoptera: Platygastridae) in soybean [21]; thrips (Thysanoptera: Thripidae) in cotton; *Gossypium hirsutum* L. [12]; and cereal leaf beetle, *Oulema melanopus* [L.] in wheat (*Triticum aestivum* L.) [22]. The spatial distributions of *S. venatus vestitus* in sod farms have not been studied. Obtaining this information could help develop an effective sampling plan and improve insecticide application strategies for *S. venatus vestitus* control.

On sod farms, *S. venatus vestitus* is managed by using insecticides [3]. Because sod farms are composed of vast land areas that are under production, application of insecticides on entire sod fields can be logistically and economically impractical in all instances; thus, growers’ resort to spotting the applications of insecticides based on the history of *S. venatus vestitus* incidence in a specific field. Here, an improved sampling method for *S. venatus vestitus* could be beneficial and will aid management decisions. Currently, there are no sampling plans for growers that guide *S. venatus vestitus* management decisions. The current plans mostly depend on visual inspections around the pavements for walking adult *S. venatus vestitus* that are conducted early in the morning, approximately one hour after sunrise. An understanding of how *S. venatus vestitus* is spatially distributed within sod fields will help to develop an effective sampling strategy using available monitoring tools.

Several geospatial methods, such as spatial analysis by distance indices (SADIE) and variograms, can be used to assess insect spatial distributions [14,20,23,24,25]. Variograms are commonly used to analyze and model the spatial dependences among individuals in a population [14,25]. Spatial dependence (or spatial autocorrelation) can be used to define the sampling scales for independent samples and to quantify the spatial patterns of insect species [11,26]. SADIE is another advanced statistical method that has been used to estimate the spatial distribution patterns of insect species based on ecological count data [14,19]. SADIE has also led to an improved understanding of pest dispersal [19,27,28], predator–prey dynamics [14,19,20], and the influence of habitat management on insect abundance [14,29]. The objective of the current study was to determine the spatial distributions of *S. venatus vestitus* in Georgia sod farms. We used variograms and SADIE to characterize the spatial distribution of *S. venatus vestitus* adults and *Sphenophorus* spp. larvae. More than 98% of *Sphenophorus* spp. adults sampled were *S.venatus vestitus,* while the remaining were *S. cariosus* Olivier and *S. inaequalis* Say. Thus, the larval samples could include other *Sphenophorus* spp. species, and there are no morphological keys available to easily distinguish billbug larvae.

## 2. Materials and Methods

### 2.1. Study Sites and General Method

Four sod fields with a history of billbug infestations in Marshallville, Georgia, USA were selected for this study. In 2019, the turfgrass genotypes in the two sod field sites were a ‘Zenith’ (Z. *japonica*) zoysiagrass (designated as site 1; 32.3843, −3.9918) and a ‘TifWay’ bermudagrass (site 2; 32.4233, −85. 8816). In 2020, the turfgrass genotypes at two different sites were ‘Zeon’ (*Z. matrella*) zoysiagrass (site 3; 32.4241, −83.8872) and ‘TifTuf’ bermudagrass (site 4; 32.4425, −83.9978).

In 2019 and 2020, *Sphenophorus* spp. larvae were sampled, whereas *S. venatus vestitus* adults were sampled only in 2020. In 2019, larval sampling of *Sphenophorus* spp. was conducted between September and December. In 2020, larvae and adults were sampled in May and June. Based on the data from Gireesh and Joseph [3], the *S. venatus vestitus* adults continuously emerge in spring and summer, which indicates the occurrence of overlapping generations in late summer and fall. Therefore, multiple stages of *S. venatus vestitus* larvae are found in the sod fields in the fall. This also suggests that the adults and various stages of *S. venatus vestitus* larvae overwinter in central Georgia. Those overwintering larvae continue to develop, and pupate and emerge as adults in late spring or summer in the following year [3]. Thus, the larval samplings conducted in fall and spring were on the same overlapping generations of *S. venatus vestitus*. For site 1, the larval samples were collected in September because the grower was harvesting sod from other areas of the field. Adult *S. venatus vestitus* were sampled only at sites 2, 3, and 4 because the sod at site 1 was harvested immediately after larval sampling. As previously described, identification of larvae at the species level is challenging and was characterized as *Sphenophorus* spp. adults were identified to the species level by using the morphological characteristics described in previous studies [30,31]. Three sites (2, 3, and 4) bordered a wood line, whereas site 1 bordered a dirt road on one side. The sod in all fields was fully grown and ready for harvest. Insecticides targeting *Sphenophorus* spp. control were not applied in 2019 and 2020 at any of the selected sites. The sites were subjected to routine mowing (twice a week), fertilizer and irrigation regimes.

### 2.2. Sampling

The sampling plan consisted of 90 sample points in a square grid, with ~3 m between any two sample points and covered a total of 27 m × 30 m (length × width) of the field. Nine sampling points for sites 2, 3, and 4 were along the X coordinate and ten points were along the Y coordinate. For Site 1, ten sampling points were along the X coordinate, and nine points were along the Y coordinate. Larval sampling for sites 1 and 2 was initiated on 30 September and 15 October 2019, respectively, and was completed on 14 December for both sites (Table 1). Larval sampling for sites 3 and 4 was conducted in 2020. For site 3, sampling was initiated on 19 May and completed on 8 June 2020. For site 4, sampling began on 1 June and ended on 8 June 2020. For larval sampling, the soil was sampled ~10 cm deep and used a 10 cm diameter Par Aide Lever Action Hole Cutter (Par Aide product company, St. Paul, MN, USA). Similarly, two more samples were obtained in the following weeks from a single sampling point (a total of three soil cores were drawn from each sampling point). These three soil samplings from a single point were 1–2 cm apart. The soil samples were transported to the laboratory in sealed plastic bags.

In the laboratory, larvae were extracted from the soil core samples contained grass roots, thatch, and soil. Adult sampling for site 2 was initiated on 26 May and completed on 8 June 2020. For site 3, sampling started on 27 May and was completed on 10 June 2020. Adult sampling for site 4 began on 10 June and ended on 24 June 2020. The adults were sampled using pitfall traps that were constructed by using 11.5-cm diameter and 7.5-cm deep clear plastic containers. The containers were partially closed with Styrofoam plates to prevent rainwater from entering the traps, and ethyl glycol was added, which acted as a preservative agent for the insects. Ninety traps were deployed at each site, and the traps were monitored weekly and were kept in place for three weeks after the date of installation. The trap contents were filtered using a sieve and were transported to the laboratory for further identification. Two geospatial methods, variograms and SADIE, were used to characterize the spatial distribution patterns of billbugs within the fields.

### 2.3. Variogram Analysis

Variograms are a commonly used method for depicting the spatial dependency of sample points. Spatial dependence is determined by developing an experimental semivariogram. Mathematically, the semivariogram (*γ*) can be represented by

$$\hat{\gamma }\left(h\right)=\frac{1}{2}\;n{\left(h\right)}_{i=1}\sum^{n\left(h\right)}z\;\left(xi\right)-z\;{\left(x_{i}+h\right)}^{2}$$

where $\hat{\gamma }$(*h*) is the estimated semivariance for the entity of interest (*z*) at all points (*x*_i_), which are separated by lag distance (*h*), and *n*(*h*) is the number of sample pairs which are separated by lag distance *h* [32].

All variogram models were created using the geostatistical software GS+ (Version 10, Gamma Design Software, LLC, Plainwell, MI, USA). Variogram models have three parameters, range, sill (C_0_ + C), and nugget (C_0_), and the values of these parameters determine the shape of the variogram. The semivariance value at which the variogram plot reaches a plateau is the sill, while the semivariance value at zero lag distance is called the nugget [33,34]. The best-fitting variogram models were used based on two criteria, the highest *γ*^2^ value or the lowest residual sum of squares (RSS) [6,35]. Curvilinear models (e.g., spherical, exponential, and Gaussian) indicate aggregation distribution patterns, which mean that neighboring sample points are spatially dependent or autocorrelated. Straight-line models (e.g., nugget and linear) represent non-aggregation or random distribution patterns with no evidence of spatial autocorrelation [33,36,37]. All models with evidence of spatial dependency have an additional parameter called “range”. Range is the maximum distance between samples below which spatial autocorrelation is present [34,38], and the range value plays a critical role in determining the adequate sampling distance for an unbiased, independent sampling plan [6,11,15,25,39]. The nugget-to-sill ratio (C_0_/C_0_ + C) and nugget were used to determine the degree of aggregation [40], where ratios <0.25, 0.25–0.75, and >0.75 indicated strong, moderate, and weak aggregation, respectively [11,41,42,43]. After selection of the variograms, interpolated pest distribution maps of billbug infestations were generated to visually demonstrate the infestation hot spots in the fields using the kriging interpolation technique [11,44,45,46].

### 2.4. SADIE Analysis

SADIE was used to characterize billbug spatial distribution patterns and test whether the resulting distributions were statistically significant [17,18]. Characterization of spatial distributions using SADIE has advantages, especially for ecological data that are collected from spatially referenced samples in which the likelihood of having zero counts at multiple sampling points is high [47,48,49]. SADIE, as an additional method, is useful for addressing some of the shortcomings of the variogram method, such as no determinations of spatial structures at low pest density with many zero counts [50].

SADIE measures the overall aggregation based on the distance to regularity (D), which represents the minimum total distance that individuals would need to move to achieve the same number (i.e., mean) for each sample point. The magnitude of D is assessed by a randomization test in which permutations of all observed counts among the sample points are performed [51]. This assessment provides an index of aggregation, *Ia*, with an associated probability, pa. Aggregated, uniform, and random distribution patterns are indicated by *Ia* > 1, *Ia* = 1, and *Ia* < 1, respectively [17]. The associated probability (i.e., *Pa* < 0.025) determines whether the resultant distribution pattern is significantly different from randomness [11,17,52]. Furthermore, mean clustering indices that represent all units in a patch are denoted by *v_i_* with an associated *p*-value, *Pv_i_*. In contrast, mean cluster indices that represent all units in a gap are denoted by *v_j_* with an associated *p*-value, *Pv*_j_. Values of *Pv_i_* and *Pv_j_* < 0.0025 indicate statistically significant gaps and patches, respectively. Calculations of the aggregation index and index of clustering in SADIE were carried out using SADIEShell (Rothamsted Experimental Station, Harpenden Herts, United Kingdom).

## 3. Results

### 3.1. Variogram Analysis

**Figure 1:**
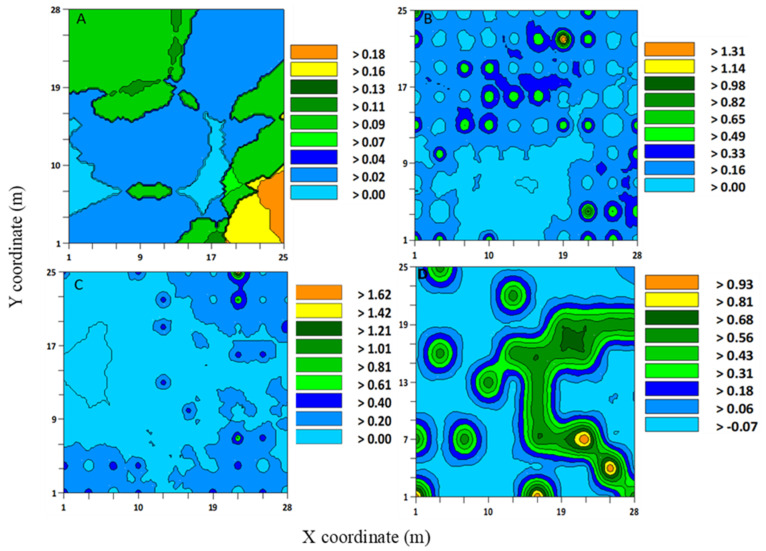
Interpolated map that was developed using the kriging based on variogram models of *Sphenophorus* spp. larvae from sod field sites (**A**) 1, (**B**) 2, (**C**) 3, and (**D**) 4 in Marshallville, Georgia (USA) in 2019 and 2020. Three-week samples were combined to form a cumulative sample at each site. Sites 1 and 2 were sampled in fall and winter 2019, and sites 3 and 4 spring 2020.

**Figure 2:**
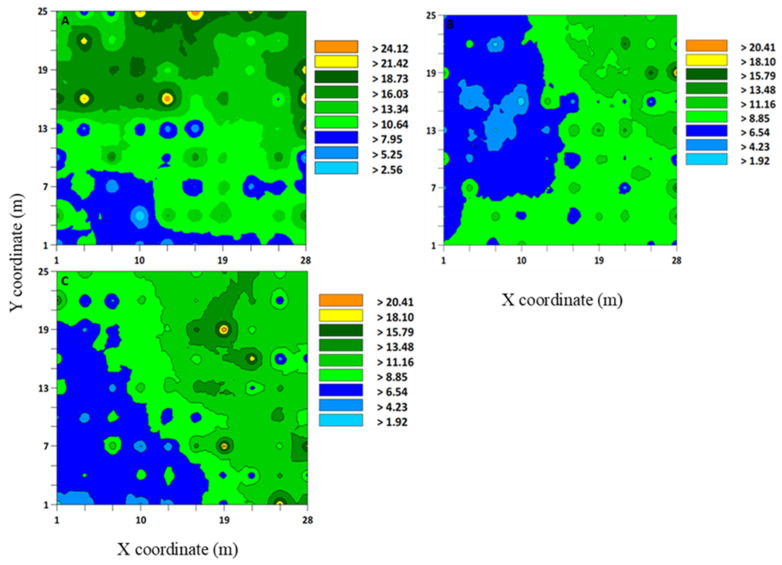
Interpolated map that was developed using the kriging based on variogram models of *Sphenophorus venatus* *vestitus* adults from three sod field sites, (**A**) 1, (**B**) 2, and (**C**) 3, in Marshallville, Georgia (USA) in 2020. At each site, three individual samples were combined (**A**–**C**).

Variogram analyses were used to evaluate spatial aggregation for *Sphenophorus* spp. larvae (Table 1, Figure 1) and *S. venatus vestitus* adults (Table 2, Figure 2).

**Figure 3:**
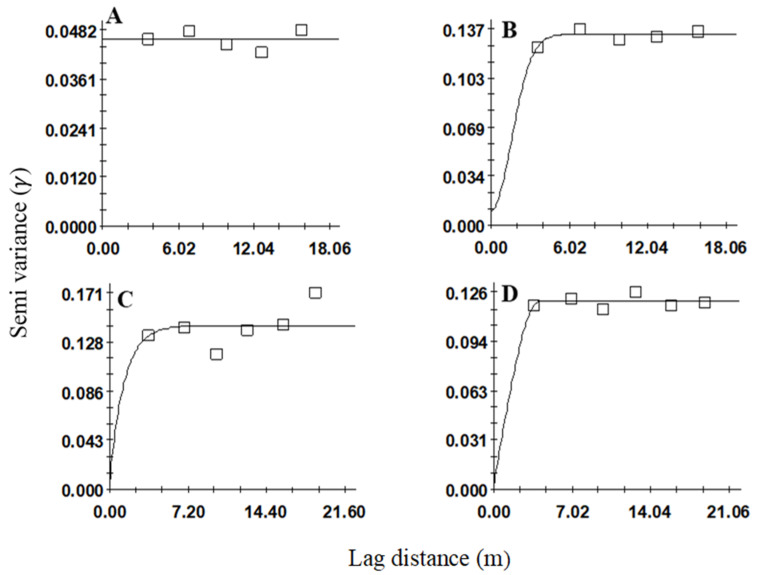
Variogram models showing the spatial distributions of *Sphenophorus* spp. larvae in sites (**A**) 1, (**B**) 2, (**C**) 3, and (**D**) 4. Sites 1 and 2 were sampled in fall and winter 2019, and sites 3 and 4 spring 2020.

The development of an omnidirectional variogram revealed aggregation patterns of larvae at three (i.e., sites 2, 3, and 4) out of the four sites. These results were based on the variogram model, high *r*^2^ and low RSS and nugget-to-sill ratio (C_0_/C_0_ + C). Based on the *r*^2^ and RSS values, the linear model fitted best for site 1 (Figure 3A), the Gaussian model for site 2 (*r*^2^ = 0.64) (Figure 3B), the exponential model for site 3 (*r*^2^ = 0.03) (Figure 3C) and the spherical model for site 4 (*r*^2^ = 0.07) (Table 1, Figure 3D). For sites 2, 3, and 4, the nugget-to-sill ratios were <0.25, which indicated strong spatial aggregation among the larval samples.

**Figure 4:**
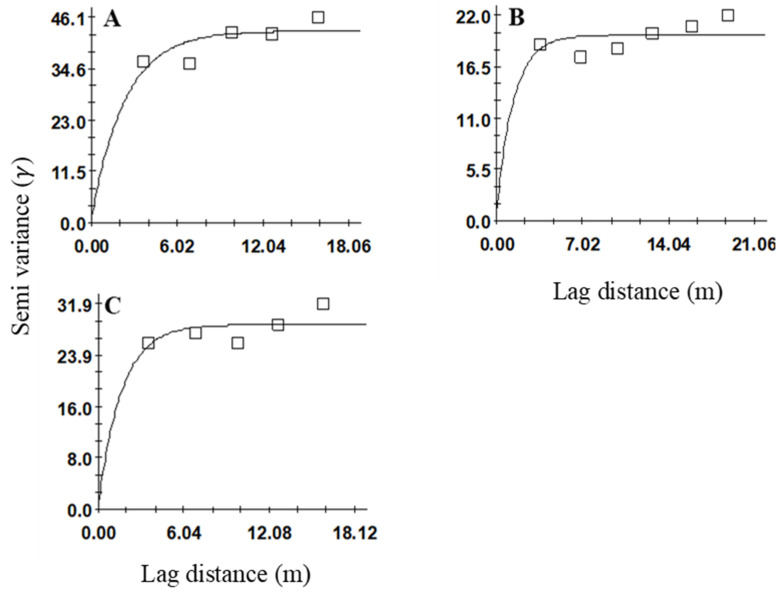
Variogram models showing the spatial distributions of *Sphenophorus venatus vestitus* adults in sites (**A**) 2, (**B**) 3, and (**C**) 4.

Spatial aggregations were observed at all three sites (2, 3, and 4) for the adult *S. venatus vestitus* populations. Variogram analyses were conducted separately on adult data for each week and cumulatively (all three weeks combined) for all three sites. For the cumulative samples, the best-fitting variogram used the exponential model at all three sites (*r*^2^ = 0.52, 0.08, 0.2 at sites 2, 3, and 4, respectively) (Table 2, Figure 4A–C). Spatial aggregation was detected in all three sampling weeks for sites 2 (*r*^2^ = 0.003, 0.5, and 0.13) and 3 (*r*^2^ = 0.24, 0.82, and 0.003), whereas at site 4, spatial aggregation was not evident in the second sample (Table 2). The best-fitting model for site 2 was the spherical model for the first week, whereas the exponential model fitted well for the two following sampling weeks (Table 2). At site 3, the best-fitting models were exponential models for all three sampling weeks. At site 4, the spherical model was the best-fitting model for the first week, whereas the linear and exponential models fitted well for the following two weeks. The nugget-to-sill ratios were <0.25, which indicated a high degree of aggregation for all three sites for the cumulative data and for all weekly sampling data for sites 2 and 3 (Table 2). For site 3, the nugget-to-sill ratio was <0.25 for the second week of sampling.

The range values that were produced by variogram analyses and indicated aggregation distributions have implications for developing sampling methods for *Sphenophorus* spp. or *S. venatus vestitus.* For *Sphenophorus* spp. larvae, the range values for these sites were between 3.82 and 4.11 m (Table 1). The interpolated maps that were developed by kriging based on selected variogram models for *Sphenophorus* spp. larvae are shown in Figure 1. For *S. venatus vestitus* adults, the range values were between 2.13 and 7.11 m (Table 2). The interpolated maps that were developed by kriging based on selected variogram models for adult *S. venatus vestitus* are shown in Figure 2.

### 3.2. SADIE Analysis

Based on the aggregation index, the spatial aggregation of *Sphenophorus* spp. larval samples were not significant for any of the three sites (Table 3). Significant spatial aggregations were observed at all three sites for *S. venatus vestitus* adult sampling (*p* < 0.025) (Table 4). The weekly analysis of *S. venatus vestitus* adult samples using SADIE detected a significant aggregation pattern in at least one of the sampling weeks for sites 2 and 4. Moreover, at site 3, significant aggregation patterns for adult *S. venatus vestitus* were observed for all three sampling weeks (Table 4).

## 4. Discussion

The spatial distribution of an insect is an inherited trait, but can be influenced by behavior and various environmental factors [8,53]. The results based on variogram and SADIE analyses showed that the adult populations of *S. venatus vestitus* followed spatially dependent distributions in the sod farms. The variogram results for *Sphenophorus* spp. larvae showed that they were spatially aggregated in 3 out of 4 sites studied. Although *S. venatus vestitus* mostly overwinter as adults [1], multiple larval stages of *S. venatus vestitus* were found in the sod farms in central Georgia. The larval stages sampled in fall or winter and spring are likely from the same overlapping generations because adult emergence was continuous from late winter to summer in the central Georgia sod farms [3]. This suggests that the distribution of larvae sampled in winter and spring in the current study is comparable and not different. Moreover, larval distribution from September samples showed no distinct pattern, possibly because of low larval densities, which affected our ability to compare differences in larval sampling in fall and spring.

A previous study showed that *S. venatus vestitus* is the dominant billbug species that causes damage (>98% of *Sphenophorus* spp. collected) in sod farms [3]. Thus, although the larval stages were not identified at the species level in the current study, they were most likely *S. venatus vestitus* larvae and, hereafter, are referred to as *S. venatus vestitus* larvae. Likewise, *S. venatus vestitus* adults were most abundant in the fully grown sod fields [3]. They frequently move from harvested to nonharvested areas of sod fields and vice versa [54]. This suggests that they are likely to colonize newly harvested sod fields and remain aggregated after the sod is harvested. These results are consistent with those for another weevil species, *L. maculicollis,* where *L. maculicollis* in golf courses was initially found to be aggregated at the edges of golf courses and then eventually dispersed throughout the entire course [19]. *L. maculicollis*, however, overwinters in leaf litter off-site and moves into golf courses during spring every year. Thus, knowledge of the aggregated distributions of *S. venatus vestitus* in sod farms will help in the development of more effective IPM.

Understanding spatial distributions helps to predict and manage pest populations by implementing accurate sampling plans and decision-making processes [25]. When using variograms to analyze the spatial distribution data, the range value of the variogram has a significant role for site-specific IPM efforts [42,55,56]. The average range value of the selected variograms in our study was 3.9 m (i.e., the cumulative mean of all three sites) for the larval *S. venatus vestitus* distributions and 5.4 m (the cumulative mean of all three sites) for the adult *S. venatus vestitus* distributions. Range values can be used either to create hotspot maps for site-specific management [57,58] or to obtain individual samples to understand the threshold values for insecticide treatments [46]. In the current study, if the range value was used for making *S. venatus vestitus* hotspot maps, the distance between two samples should be less than 3.9 m and 5.4 m for larvae and adults, respectively. Hotspot maps indicate those areas with high degrees of infestation and therefore, they help with information-based decision-making for pest management [57]. However, developing distribution maps may not be feasible for sod growers because they require many sample points and substantial technical skills to process the raw data for map construction [20,59]. When the range values are used to obtain unbiased samples, the distances between two sampling points should be greater than the average range values for both larvae and adults [15,26,58]. *S. venatus vestitus* larvae are hidden in the soil, and thus, their infestations in soils can be determined if soil samples are collected using a hole cutter at 4.0 m (average range value = 3.9 m) distances to capture larvae. Because the larval samples were mainly collected in winter and spring, further research is warranted to determine the larval distribution of *S. venatus vestitus* in summer and early fall. Similarly, the prevalence of *S. venatus vestitus* adults can be determined if they are collected by deploying pitfall traps at 5 m (the average range value = 4.7 m) distances at 7 d intervals. This information can be used for pest management decisions.

The variogram and SADIE analyses showed inconsistent results, in which both *S. venatus vestitus* larvae and adults showed aggregations in the variogram analyses. However, only for *S. venatus vestitus* adults did the data support aggregation when using SADIE analysis. This discrepancy may be due to the variations in which the spatial weights are calculated for individual sample points [60,61]. SADIE measures spatial dependence based not only on relative positions but also on the absolute sampling positions of the counts [22,62]. As a result, spatial aggregation is sometimes not observed due to the higher values of isolated individual sampling points. In contrast, variogram analysis includes these higher values, which can contribute to the aggregated distribution patterns of insect populations [6,26]. Therefore, the use of more than one geospatial technique is preferred to address this discrepancy between methods when determining spatial distribution patterns [59]. A previous study combined variogram and SADIE to generate prescription maps for the bean leaf beetle, *Cerotoma trifurcata* Forster (Coleoptera: Chrysomelidae) [63]. Another study used semivariograms and SADIE to understand the spatiotemporal patterns of *Ricania shantungensis* (Hemiptera: Ricaniidae) in chestnut fields [58]. A previous study investigated and differentiated various statistical methods and found that no single method could completely identify all spatial characteristics of the dataset [49,60]. Moreover, combining both global and local methods provide clarity for various aspects of spatial patterns and thereby provides an exact elucidation of spatial heterogeneity [60,61,64]. While variograms revealed the spatial dependences among the larval and adult populations in our study, SADIE detected significant aggregation patterns only for adults. Similar discrepancies in results when using variograms and SADIE have been reported in previous studies [11,59]. The main aim of combining several geospatial methods should be to provide better accuracy of results and not to validate the results of one method over the other [49,64].

This is the first study that shows the spatial distributions of *S. venatus vestitus* in sod farms. Although most previous studies have used adult *S. venatus vestitus* sampling, larval sampling remains challenging [4,5,31]. Moreover, in sod farms and golf courses, *S. venatus vestitus* larvae cause more economic damage than adults [5]. Thus, understanding the distribution patterns of *S. venatus vestitus* larvae is critical to developing an effective strategy for addressing this pest from a management standpoint. The current practice of larval sampling using hole cutters requires more labor and time and has still not been demonstrated to be an efficient method for determining *Sphenophorus* spp. larvae distributions [5]. Based on our study, we suggest that soil samples using a 10-cm hole cutter should be taken 4.0 m apart at ~10 cm depth to indicate the prevalence of aggregated patches of *S. venatus vestitus* larvae in the field, especially in the winter and spring months. Further research is warranted to determine the minimum number of samples per sod field to quantify *S. venatus vestitus* and develop thresholds for management decisions. This information can be used for spot applications or site-specific management of *S. venatus vestitus* larvae and *S. venatus vestitus* adults in sod farms and can reduce insecticide use and application costs.

## Figures and Tables

**Table 1:** Variogram models and parameters representing the spatial distribution patterns of *Sphenophorus* spp. larvae at four sites in Marshallville, Georgia (USA) in fall and winter 2019 and spring 2020.

Site ^†^	Sampling Time	Range (m) ^‡^	Model ^§^	*r* ^2^	C_0_ ^‡^	C_0_ + C ^‡^	C_0_/C_0_ + C ^‡^
**1**	Fall	− ^¶^	Linear	0.003	−	−	−
**2**	Winter	3.82	Gaussian	0.64	0.009	0.133	0.060
**3**	Spring	3.9	Exponential	0.03	0.003	0.142	0.020
**4**	Spring	4.11	Spherical	0.07	0.001	0.120	0.008

Site ^†^ 1, ‘Zenith’ zoysiagrass (*Z. japonica*); Site 2, ‘TifWay’ bermudagrass (*Cynadon* spp.); Site 3, ‘Zeon’ zoysiagrass (*Z. matrella*); and Site 4, ‘TifTuf’ bermudagrass (*Cynadon* spp.). Three-week samples were combined to form a cumulative sample at each site. ^‡^ variogram parameters; range, nugget (C_0_), sill (C_0_ + C), and nugget-to-sill ratio (C_0_/C_0_ + C). ^§^ Spherical and exponential are curvilinear models (indicating aggregation distribution) Linear, and straight-line models (aggregation not observed). ^¶^ Aggregation not observed.

**Table 2:** Variogram models and parameters representing the spatial distribution patterns of *Sphenophorus venatus vestitus* adults at three sites in Marshallville, Georgia (USA), in 2020.

Site ^†^	Date	Range (m) ^‡^	Model ^§^	*r* ^2^	C_0_ ^‡^	C_0_ + C ^‡^	C_0_/C_0_ + C ^‡^
2	26 May	3.50	Spherical	0.000	0.001	0.600	0.001
	2 June	3.39	Exponential	0.500	0.650	14.80	0.040
	9 June	3.72	Exponential	0.130	1.310	16.78	0.070
	Combined ^†^	7.11	Exponential	0.529	1.300	43.09	0.030
3	27 May	6.50	Exponential	0.240	0.040	7.760	0.005
	3 June	4.50	Exponential	0.820	0.010	4.970	0.002
	10 June	2.13	Exponential	0.030	0.001	0.630	0.001
	Combined	4.2	Exponential	0.080	1.060	19.02	0.050
4	10 June	3.72	Spherical	0.947	0.030	16.19	0.001
	17 June	- ^¶^	Linear	0.101	-	-	-
	24 June	5.97	Exponential	0.828	0.060	0.700	0.080
	Combined	4.98	Exponential	0.200	0.690	28.73	0.020

Site ^†^ 2, ‘TifWay’ bermudagrass (*Cynadon* spp.); Site 3, ‘Zeon’ zoysiagrass (*Z. matrella*); and Site 4, ‘TifTuf’ bermudagrass (*Cynadon* spp.). Three-week samples were combined to form a cumulative sample at each site. ^‡^ variogram parameters; range, nugget (C_0_), sill (C_0_ + C), and nugget-to-sill ratio (C_0_/C_0_ + C). ^§^ Spherical and exponential are curvilinear models (indicating aggregation distribution) Linear, and straight-line models (aggregation not observed). ^¶^ Aggregation not observed.

**Table 3:** Parameters for the spatial distribution patterns of *Sphenophorus* spp. larvae using SADIE at four sites in Marshallville, Georgia, in 2019 and 2020. Sites 1 and 2 were sampled in fall and winter 2019, and sites 3 and 4 spring 2020.

Site ^†^	*Ia* ^‡^	*PIa* ^‡^	*v_j_* ^§^	*v_i_* ^¶^	*Pv_j_* ^§^	*Pv_i_* ^¶^
1	1.153	0.157	−1.157	1.073	0.154	0.272
2	0.900	0.715	−0.899	0.934	0.713	0.610
3	1.070	0.304	−1.063	1.024	0.326	0.387
4	1.015	0.386	−1.019	1.056	0.396	0.304

Site ^†^ 1, ‘Zenith’ zoysiagrass (*Z. japonica*); Site 2, ‘TifWay’ bermudagrass (*Cynadon* spp.); Site 3, ‘Zeon’ zoysiagrass (*Z. matrella*); and Site 4, ‘TifTuf’ bermudagrass (*Cynadon* spp.). Three-week samples were combined to form a cumulative sample at each site. *Ia* ^‡^; index of aggregation, *PIa* ^‡^; with an associated probability, significant aggregation at *p* < 0.025; *v_j_* ^§^; mean clustering indices representing all units in a patch, *Pv_j_* ^§^, with an associated probability. *v_i_* ^¶^; mean cluster indices representing all units in a gap, *Pv_i_* ^¶^, with an associated probability.

**Table 4:** Parameters for the spatial distribution patterns of *Sphenophorus venatus vestitus* adults using SADIE at three sites in Marshallville, Georgia (USA) in 2020.

Site ^†^	Date	*Ia* ^‡^	*PIa* ^‡^	*v_j_ ^§^*	*v_i_* ^¶^	*Pv_j_* ^§^	*Pv_i_* ^¶^
2	26 May	1.613	0.002 **	−1.572	1.609	0.003 **	0.002 **
	2 June	1.283	0.051	−1.236	1.286	0.083	0.051
	9 June	1.105	0.215	−1.118	1.146	0.208	0.158
	Combined ^†^	1.740	<0.001 **	−1.864	1.699	<0.001 **	0.001 **
3	27 May	1.842	<0.001 **	−1.723	1.757	<0.001 **	<0.001 **
	3 June	1.456	0.014 *	−1.332	1.472	0.030 **	0.005
	10 June	1.864	<0.001 **	−1.837	1.573	<0.001 **	0.004 **
	Combined	1.518	0.007 **	−1.509	1.587	0.006 *	0.003 **
4	10 June	1.071	0.266	−1.084	1.055	0.238	0.279
	17 June	1.379	0.027	−1.438	1.204	0.011 *	0.084
	24 June	1.286	0.056	−1.358	1.176	0.026	0.118
	Combined	1.728	0.001 **	−1.612	1.451	0.002 **	0.011 *

Site ^†^ 2, ‘TifWay’ bermudagrass (*Cynadon* spp.); Site 3, ‘Zeon’ zoysiagrass (*Z. matrella*); and Site 4, ‘TifTuf’ bermudagrass (*Cynadon* spp.). Three-week samples were combined to form a cumulative sample at each site. *Ia* ^‡^; index of aggregation, *PIa* ^‡^, with an associated probability, significant aggregation at *p* < 0.025. *v_j_* ^§^; mean clustering indices of all units in a patch, *Pv_j_* ^§^, with an associated probability. *v_i_* ^¶^; mean cluster indices of all units in a gap, *Pv_i_* ^¶^, with an associated probability. * Significant at *p* ≤ 0.025, ** Significant at *p* ≤ 0.005.

## Data Availability

Not applicable.
